# The Development of Integrated Stroke Care in the Netherlands a Benchmark Study

**DOI:** 10.5334/ijic.2444

**Published:** 2016-11-16

**Authors:** Lidewij E. Vat, Ingrid Middelkoop, Bianca I. Buijck, Mirella M.N. Minkman

**Affiliations:** NL SUPPORT, Memorial University Newfoundland, CA; Stroke Knowledge Network of the Netherlands, NL; Erasmus MC University Medical Center/Rotterdam Stroke Service, NL; Vilans, The National Center of Expertise in Long-Term Care, Catharijnesingel 47, 3503 RE Utrecht, The Netherlands, NL

**Keywords:** integrated care, stroke services, development, quality management

## Abstract

**Introduction:**

Integrated stroke care in the Netherlands is constantly changing to strive to better care for stroke patients. The aim of this study was to explore if and on what topics integrated stroke care has been improved in the past three years and if stroke services were further developed.

**Methods:**

A web based self-assessment instrument, based on the validated Development Model for Integrated Care, was used to collect data. In total 53 coordinators of stroke services completed the questionnaire with 98 elements and four phases of development concerning the organisation of the stroke service. Data were collected in 2012 and 2015. Descriptive-comparative statistics were used to analyse the data.

**Results:**

In 2012, stroke services on average had implemented 56 of the 89 elements of integrated care (range 15–88). In 2015 this was increased up to 70 elements on average (range 37–89). In total, stroke services showed development on all clusters of integrated care. In 2015, more stroke services were in further phases of development like in the consolidation and transformation phase and less were in the initiative and design phase. The results show large differences between individual stroke services. Priorities to further develop stroke services changed over the three years of data collection.

**Conclusions:**

Based on the assessment instrument, it was shown that stroke services in the Netherlands were further developed in terms of implemented elements of integrated care and their phase of development. This three year comparison showed unique first analyses over time of integrated stroke care in the Netherlands on a large scale. Interesting further questions are to research the outcomes of stroke care in relation to this development, and if benefits on patient level can be assessed.

## Introduction

In the Netherlands, health care professionals and organisations are challenged to provide high quality health and social care. It is their ambition to offer long-term care services and welfare to an aging population in a patient centered and cost-efficient manner. To face this challenge, a number of reforms and new policies are implemented during the last years. These new policies focus on an increased responsibility for care and welfare on the decentralized level of the municipality. Another change is the introduction of (local) district nurses and transferring more responsibilities to civilians and local communities themselves [1,2]. Within this context, integrated care networks for the elderly and the chronically ill have to deal with new regulations, a diversity of stakeholders and different organisational models.

In the Netherlands, individuals who need integrated care, such as stroke patients, receive integrated care in collaborative networks of health and social care providers. In the Netherlands, every year 47,000 people suffer from a stroke: a Cerebral Vascular Accident (CVA) [3]. Currently, there are about 240.000 patients that have had a stroke [4]. In the next decade, the number of stroke patients is expected to increase. Strokes cause the third biggest burden of disease within the Dutch population [5], responsible for 2.5% of its total health care costs [6]. In 2000, roughly 50% of first stroke patients died within 12 months after hospitalization. By 2005, this has been reduced to 22% partly due to more attention for aftercare by stroke services [7].

A stroke service can be defined as a network of providers working together during the acute, the rehabilitation and the chronic phase of stroke patient care [8]. A large number of disciplines and organisations such as hospitals, nursing homes, rehabilitation centers, general practitioners and home care providers, are involved in the provision of stroke care. Stroke services aim to deliver coherent and patient centered integrated care [9]. This requires a regional setting with all relevant health and social care stakeholders and the local community, working together to provide multidisciplinary, coordinated care and support [10]. Currently, there are approximately 75 stroke services in the Netherlands [11].

The organisation of integrated stroke care is one of the oldest initiatives of integrated care in the Netherlands. Integrated stroke care started its development in the nineties with the organisation of specific stroke units in hospitals and nursing homes. Next steps were further development into integrated stroke networks or services. This development was stimulated by the Dutch Heart Association and a number of national initiatives [8,12]. A number of innovations such as the development of care pathways, indicator frameworks and care standards followed. During the last decade there has been a focus on continuous development and further improvement of integrated stroke services. This resulted in more coherent care for stroke patients, increased satisfaction among patients and caregivers, and also leading to more cost-effective care [13]. The already long history of integrated stroke care and the results that are achieved makes it an interesting object of study.

In 2006 the Stroke Knowledge Network of the Netherlands (“Kennisnetwerk CVA Nederland”) was founded by professionals and coordinators in stroke care. One of the motives for establishing this network was the declaration of the ‘Helsingborg Consensus Conference on European Stroke Strategies’. The declaration describes the aims and goals to be achieved by the year 2015 like the development of a system of routine data collection necessary to evaluate the quality of stroke care [14,15]. In this perspective and because the network strives to facilitate improvements over time, the network was searching for a conceptual framework that could help them to assess and improve the organisation of integrated stroke care. The network adapted the Development Model for Integrated Care as their framework for this purpose [16].

The aim of this study was to explore the development of stroke services in the Netherlands over time by using a self-evaluation instrument which was based on the Development Model for Integrated Care. A comparison over time was needed to assess if stroke services improved their integrated stroke care for patients by the year 2015 compared to the situation three years earlier. The goal was to stimulate and facilitate knowledge exchange and improvement between stroke services and their coordinators. The results of a benchmark study between 2012 and 2015 are presented in this article.

## Theory and methods

A digital web-based self-evaluation tool (questionnaire) for integrated care services based on the Development model for Integrated Care was developed. The tool gives integrated care services the possibility to evaluate their development and identify activities for improvement. The Stroke Knowledge Network Netherlands added nine stroke specific elements to the original 89 elements of the model. The Stroke Knowledge Network Netherlands offers their member’s two-yearly the opportunity to evaluate their service by using the web-based self-evaluation tool. In 2012 and 2015, 75 coordinators of strokes services received an invitation by the Stroke Knowledge Network Netherlands to evaluate their integrated care service by filling in the web-based questionnaire. Participation was voluntary. All participants received a personal code to enter the self-evaluation tool. Data were kept confidential and were not shared with other organisations.

The stroke services received a short instruction about the tool by email. The self-evaluation tool exists of three parts: A, B and C. In part A the respondents were asked to provide general information about their stroke services. In part B each respondent rated the presence of all elements in their integrated care service and indicated which elements were priorities for the coming year. If it was not clear if an element had been implemented, this could be rated as ‘unknown’. In part C the coordinators were asked to estimate in which phase of development they would self–assess their service. All coordinators were asked to fill in one questionnaire on behalf of the total integrated care service. They could discuss the questions with stakeholders or complete the questionnaire by themselves. This was up to the coordinators.

The same person completed the questionnaire on both occasions. The stroke services differed in their demographic area (urban, rural) and economic context (related insurance company). They had a similar political context (policy, health care legislation).

### Conceptual model

**Figure 1:**
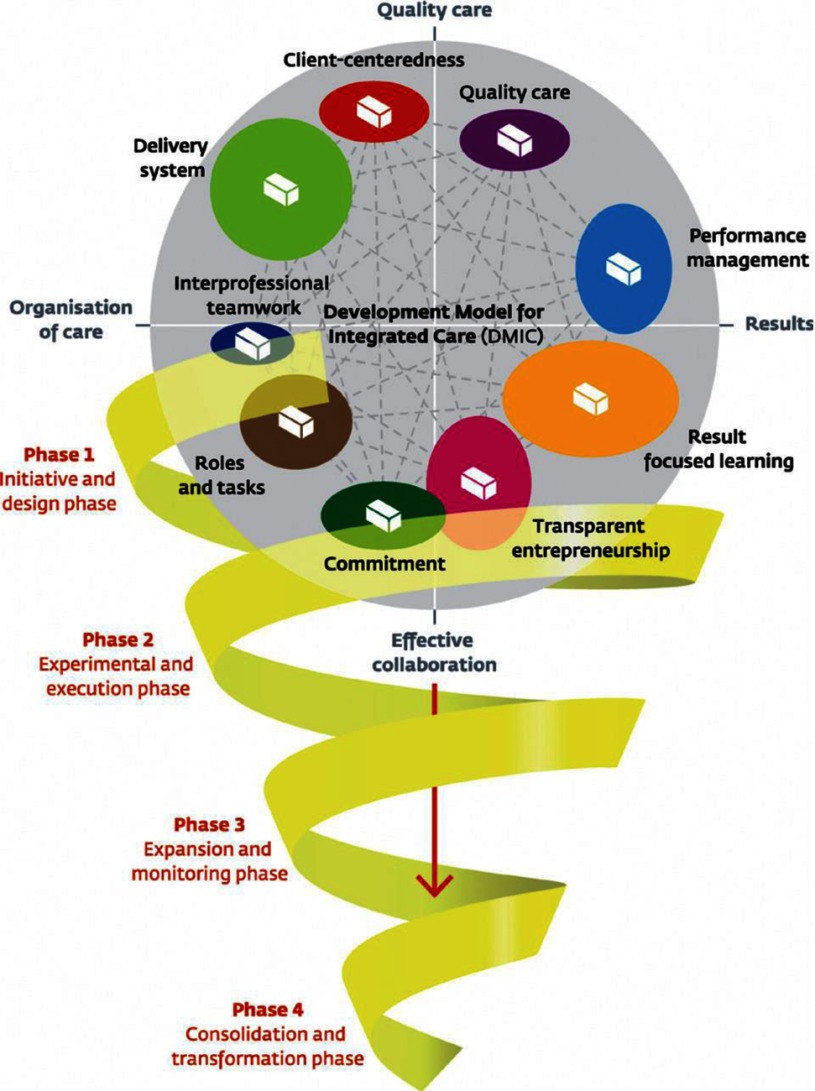
Development Model for Integrated Care.

The Development Model for Integrated Care is systematically developed based on a literature study, a Delphi study and multiple validation studies (Figure 1). Eventually 89 unique elements of integrated care were determined, which were grouped into nine clusters. The clusters are named: ‘client-centeredness’, ‘delivery system’, ‘performance management’, ‘quality of care’, ‘result-focused learning’, ‘interprofessional teamwork’, ‘roles and tasks’, ‘commitment’, and ‘transparent entrepreneurship’ [16,17]. Elements of integrated care are described in terms of activities that can be undertaken to implement and develop integrated care. Some examples are: ‘Using a protocol for the systematic follow-up of patients’; ‘using common care and treatments plans across the entire care continuum’; ‘reaching agreements on each care partners specific areas (who does what)’ or ‘stimulating trust among care partners’ and ‘using self-management support methods as a part of integrated care’. Furthermore, the Development Model for Integrated Care describes four phases of development: the initiative and design phase; the experimental and execution phase; the expansion and monitoring phase; and the consolidation and transformation phase (Table 1). Next to a description of phase characteristics, for each phase the top 10 of most relevant elements for that phase were determined in previous research [18].

**Table 1:** Development Model for Integrated Care – Description of development phases.

**PHASE 1 Initiative and design phase:** The collaboration between health care providers has been intensified or started up. The starting point is a common problem or chance occurrence, or builds on current cooperation among care professionals. There is a sense of urgency and there are possibilities for working on these challenges in collaboration. The targeted patient group, the care chain and care process have been defined, as also the needs of patients and stakeholders. The level of ambitions, motivation and leadership determine the progress achieved. A multidisciplinary team designs an experiment or project to execute the current ideas. The collaboration can be signed up to in an agreement among care partners. *Keywords*: Exploring possibilities/impossibilities, ambitions and chances, (project) design and collaboration agreements.
**PHASE 2 Experimental and execution phase:** New initiatives or projects are being executed in the care chain. The aims, content, roles, and tasks in the care chain have been clarified and written down in care pathways and protocols. There is coordination at the level of the care chain by for instance installing coordinators or setting up meetings. Information about patient groups, working procedures or professional knowledge is exchanged. There are experiments within the collaboration, results are evaluated to learn from and reflect on. Preconditions for projects have been considered and boundary conditions have been solved by collaborative means or agreements among care providers. *Key words*: Writing down aims and content of the collaboration, coordination at care chain level, experimenting and reflecting.
**PHASE 3 Expansion and monitoring phase:** Projects have been expanded or integrated in integrated care programs. Agreements on the content, tasks and roles within the care chain are clear and signed up. Collaboration is no longer on an informal basis. Results are systematically monitored and improvement areas identified. The targeted population has been surveyed. More collaborative initiatives emerge such as mutual education programs. There is a continuous commitment to the ambition of the integrated care program. Interorganisational barriers and fragmented financial structures are on the agenda of the care partners. *Keywords*: Further development and maturity, monitoring and improving results, new questions and innovation.
**PHASE 4 Consolidation and transformation phase:** The integrated care program is the regular way of working and providing care. Coordination at care chain level is operational; information is shared, transferred and fed back. A monitoring system periodically shows if results are being sustained, what specific improvement possibilities have been identified and to what extent patient needs have been met. The program builds further on successful results. Organisational structures transform or are newly designed around the integrated care program. Financial agreements are arranged with financers by means of integral contracts covering the care chain as a whole. Partners in the care chain explore new options for collaboration in the external environment with other partners. *Keywords*: Continuous improvement, new ambitions, structures fitting the integrated care program (organisational structures, integral financing).

The model is validated in practice by assessing the relevance and implementation of the elements and development phases in 84 integrated care services in The Netherlands: in stroke, acute myocardial infarct (AMI), and dementia services [18,19]. Recently, a Canadian study was published which used the model as well [20].

### Data analysis

Data were analysed by the research team which consisted of the coordinator of the Stroke Knowledge Network and researchers of Vilans, The National Center of Expertise in Long-Term Care. First of all, the characteristics of the stroke services were described using an average- and multiple response analysis. Secondly, the number of implemented integrated care elements and priorities per stroke service were analysed using descriptive-comparative analysis. Thirdly, the cluster scores per year were calculated by an average analysis based on the total number of implemented elements per cluster divided by the total number of stroke services. The scores were converted into percentages and were not adjusted for the number of elements per cluster.

Furthermore, data on the phase of development were analysed. Based on the self-assessed presence of elements, the phase of development was determined by using the top-10 of the Development Model for Integrated Care. In case that seven or more elements of the phase specific top ten were assessed as implemented, the phase was rated as completed. These cut-off points were developed in previous research [21]. The calculated phase of development was compared to the assessed phase of development. In addition, the results of 2012 were compared to the results of 2015 to analyse changes over time. For both moments of data collection, the most implemented and most prioritized elements between 2012 and 2015 were analyzed by using descriptive statistics.

## Results

In 2012, 67 stroke services out of 75 evaluated their integrated care service (response 89%). Three years later, in 2015, 59 stroke services (re-)evaluated their services based on the Development Model for Integrated Care (response 79%). Those that evaluated their services in both 2012 and 2015 were included in this study (n=53).

### Characteristics

The characteristics of the stroke services are presented in Table 2. In 2015 the stroke services existed approximately 12 years. There is variation in the age of stroke services; some of them started recently while others have been collaborating for many years. Furthermore, there is a difference in the numbers of stroke patients and partners per stroke service. By 2015, all stroke services had a coordinator. The amount of hours that a coordinator was formally scheduled to spend on integrated care development ranged from 0 hours per week up to 24 hours per week. Data collection and partner meetings slightly increased between 2012 and 2015, whereas agreements with health insurance companies decreased.

**Table 2:** Characteristics of stroke services (2012, 2015).

Characteristics stroke services	2012 (n = 53, 100%)	2015 (n = 53, 100%)

Age	Average 9 years (range 0 – 17)	Average 12 years (range 3 – 20)
Total care provider organisations	Average 7 (range 2 – 19)	Average 7 (range 2 – 19)
Number of stroke patients last year	Average 345 (range 120 – 983)	Average 492 (range 79 – 1650)
Background of members in workgroups	Only managers: 4% Only professionals: 13% Both: 70% No workgroups: 13%	Only managers: 0% Only professionals: 17% Both: 78% No workgroups: 5%
Coordinator	Yes: 92% Average 7,5 hours per week (range: 0 – 24)	Yes: 100% Average 9 hours per week (range: 0 – 24)
Signed agreement of collaboration between providers of the stroke services	Yes: 81%	Yes: 81%
Regular meetings with partners of the stroke network	Yes: 77%	Yes: 91%
Data collection on indicators	Yes: 87%	Yes: 99%
Agreement with healthcare insurance company	Yes: 55%	Yes: 51%

### Integrated care elements

**Figure 2:**
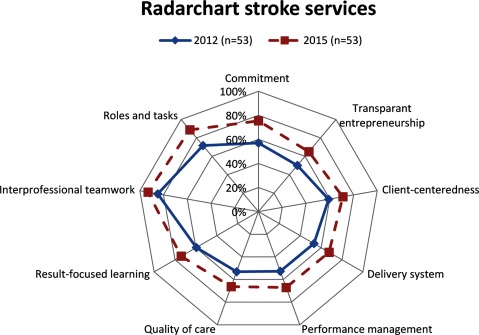
Average percentages of implemented integrated care elements per cluster 2012 (blue line) and 2015 (red dotted line).

In 2012, stroke services on average had implemented 56 of the 98 elements of integrated care. In 2015 this increased up to 70 elements. The minimum and maximum scores ranged between 15 elements and 88 elements in 2012, and 37 elements and 89 elements in 2015. The average percentage of present integrated care elements as indicated by the 53 coordinators is presented in a radar chart (Figure 2). Overall, the radar chart shows that stroke services have implemented more elements in 2015 than the three years prior. On average, stroke services showed development on all clusters of integrated care.

### Development phases

**Figure 3:**
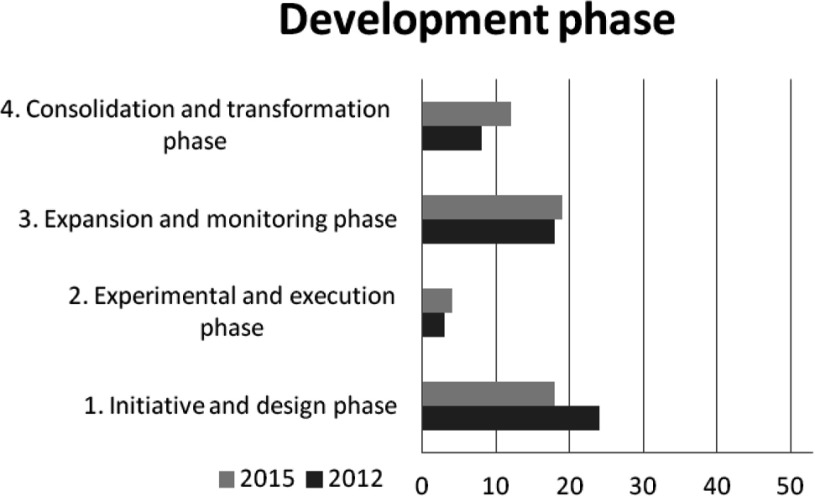
Stroke services per phase of development based on the Development Model for Integrated Care in 2012 (black line) and 2015 (grey line).

Figure 3 shows the distribution of stroke services at the different phases of development based on the Development Model for Integrated Care. On average there are more stroke services in the consolidation and transformation phase (phase 4) and less in initiative and design phase (phase 1) in 2015 than in 2012. The amount of stroke services in the experimental and execution phase (phase 2) and the expansion and monitoring phase (phase 3) showed small differences. The results show a difference in development per stroke service. A large number of the stroke services (n=20) were in the similar development phase in 2015 as in 2012. Others developed one (n=9), two (n=10) or three (n=3) phases. In 2015, a few stroke services showed one (n=3), two (n=4) or three (n=4) phases of regression compared to 2012.

The assessed development phases by the coordinators in 2015 partly overlap the results of the Development Model for Integrated Care. Most coordinators (n=31) assessed their service in 2015 in phase 3. Twenty coordinators assessed their phase equal to the Development Model for Integrated Care in 2015. Twenty-one coordinators estimated their stroke services were one, two or three phases higher whereas twelve coordinators estimated their stroke services were one phase lower compared to the results of the Development Model for Integrated Care in 2015.

### Differences over time

Table 3 shows the top-10 most implemented elements in 2012 and 2015. Comparably, six elements of the ten elements of 2015 are similar to the top-10 of 2012. Compared to 2012, more stroke services implemented the elements listed in the top 10.

**Table 3:** Top-10 of most implemented elements in 2012 and 2015.

Top-10	Elements most implemented in 2012	N (100% = 53)	Elements most implemented in 2015	N (100% = 53)

1.	Being a member of the Stroke Knowledge Network Netherlands	53	Being a member of the Stroke Knowledge Network Netherlands	53
2.	Organising a 24-hour availability for thrombolysis in the care chain (7 days a week)	50	Working in multidisciplinary teams	53
3.	Defining the targeted patient group	49	Directing the care chain by appointing a limited number of people with coordinating tasks	53
4.	Working in multidisciplinary teams	49	Organising a 24-hour availability for thrombolysis in the care chain (7 days a week)	52
5.	Installing a coordinator working at the chain-care level	46	Defining the targeted patient group	52
6.	Achieving adjustments among care partners by means of direct contact	46	Installing a coordinator working at the chain-care level	52
7.	Reaching agreements on referrals and the transfer of patients through the care chain	46	Involving leaders in improvement efforts in the care chain	52
8.	Delivery of indicator data of the chain to the benchmark of the Stroke Knowledge Network Netherlands	46	Delivery of indicator data of the chain to the benchmark of the Stroke Knowledge Network Netherlands	51
9.	Using evidence-based guidelines and standards	45	Assuring the leadership commitment of the partners involved in the care chain	51
10.	Reaching agreements on chain logistics (e.g. waiting periods and throughput times)	45	Striving toward an open culture for discussing possible improvements for care partners	51

Table 4 shows the top-5 elements that had been most prioritized in 2012. One of the most prioritized items has been substantially more implemented in 2015. One of the elements ‘Using a single patient-monitoring record accessible to all care partners’ was less implemented within three years, whereas ‘connections with databases of partners in the care chain’ has been more often implemented. ‘Monitoring of patient judgements and satisfaction for the whole care chain’ has barely been increased in 3 years. Table 5 shows the elements that had the highest implementation rate between 2012 and 2015.

**Table 4:** Most prioritized elements of 2012.

Top-5	Most prioritized in 2012	Difference in implementation (n = n2015 – n2012)

1.	Monitoring patient judgements and satisfaction for the whole care chain	4
2.	Developing connections with the databases of partners in the care chain	7
3.	Developing a multidisciplinary care pathway	13
4.	Using a single patient-monitoring record accessible to all care partners	–3
5.	Collecting patient feedback and patient experiences for improving the care chain	7

**Table 5:** Elements with highest implementation rate between 2012 and 2015.

Top-5	Highest implementation rate 2015	Difference in implementation (n = n2015 – n2012)

1.	Using uniform patient-identification numbers within the care chain	20
2.	Reaching consensus about partner domains	19
3.	Gathering patient-related performance data (health status, quality of life)	19
4.	Describing the tasks and authorities of leaders, coordinators and advisory boards in the care chain	18
5.	Attention to connect the care chain to house-, welfare- and work domains	18

Stroke service coordinators received a benchmark report of the total group (anonymously reported) and an individual report. The coordinators were stimulated to use these reports and the digital tool to discuss the results with their local partners in order to reflect on their situation and define improvements for their patients. The Stroke Knowledge Network facilitated this process.

## Discussion and conclusion

The results of this benchmark study show that stroke services implemented more elements of integrated care in the past three years. On average, stroke services show development on all clusters of the model covering a diversity of aspects of integrated care. Related to this, the results show that after three years there are more stroke services in further phases of development like the consolidation and transformation phase and fewer services are in the earlier phases like the initiative and design phase. Overall, stroke services in the Netherlands seem to have worked actively on realizing integrated care for their patients.

Stroke services differ in their characteristics like size, number of patients, involved health and social care providers and in their focus. New collaborations did emerge, but overall they worked on further development of their stroke service. Stroke services prioritized various elements over time. They prioritized elements from several phases of the Development Model of Integrated Care, although the model recommends focusing on the top-10 elements per phase before continuing with the next phase. However, the most prioritized elements correlated with the expansion and monitoring phase (phase 3), in which most stroke services assessed their services. Of the most prioritized elements to implement in 2012, only a few were frequently implemented during the years afterwards. Overall the number of implemented elements did increase, but probably other factors like changes in context, policy, treatment possibilities, or the role of leaders and coordinators might eventually determine were the focus is on. For example, new regulations were implemented in 2015. These new policies focus on an increased responsibility for care and welfare on the decentralized level of the municipality. Therefore, it’s understandable that the attention to connect the care chain to house-welfare and work domains was reflected in the results. Another issue in this perspective is the high number of different stakeholders and professionals in stroke services. How to look at an integrated care initiative, how to assess or judge it and determine what is important seems also related to the background of these stakeholders. A benchmark study in Dutch diabetes care showed that the assessment of their diabetes network by the involved stakeholders varied between stakeholders and was related to their background and role. Coordinators and stakeholders who had a more generic or managerial role seem to score more aspects of integrated care as present than stakeholders with a more professional background [22]. A Canadian study analyzed the implementation and development of integrated care initiatives for patients with chronic obstructive pulmonary disease, mental health problems and for palliative pathways. In this study the Development Model for Integrated Care was also used as the evaluation framework. They found that the background of nurses was related to how they assessed their integrated care, with nurses with coordinative roles ranking higher scores. Besides this background perspective which has to be taken into account, the Canadian study also stated that the context like changing policy or legislation played an important role in the development of these programs [20]. In our case of the stroke services, this could also have been an important factor. The Dutch health care system and especially long term care is in transition, which has an impact on multiple levels [1,2].

Another finding is that only a few coordinators assessed their stroke service in the first phase of development, the initiative and design phase. However, based on the phase scores calculated by the Development Model for Integrated Care about one third of the integrated care services are positioned in this first phase in 2015. This is an interesting finding because stroke services exist relatively long, being one of the first patients groups where local collaboration in networks was realized on a large scale. There could be multiple considerations for this finding. First, earlier work shows that developing integrated care is a dynamic long-term process in which steps forward can be followed by steps backwards and so forth. In dynamic periods, reconsidering the aims and focus of the collaboration which is important in the first phase can be relevant again. Another issue could be the possible bias by background as described above, because scoring the phase of development was merely done by coordinators. Coordinators scored a phase based on their judgement when reading text about each phase, whereas the Development Model for Integrated Care phase scores were calculated based on the scores on each element in the questionnaire. Coordinators being a central figure in stroke services; they have a good overview of the stroke service and are well informed about issues that occur and agreements that are made. On the other hand, this could also be a bias because they could be more informed than their partners in the service and therefore be more (or too) optimistic. In a recent diabetes benchmark study [22] higher scores of coordinators were found compared to their partners (for instance professionals) in their integrated care service. Another issue that could play a role are the (necessary) cut-off points in the analyses whereas seven or more elements have to be present to consider a phase as ‘completed’. In practice development is dynamic and often also multiple elements of following phases are already present which influences the self-assessment. Similar results have been found for other integrated care services [20,22].

Based on the results of this study it can be concluded the priorities of integrated care services changed over the three years. More attention was given to using uniform patient-identification numbers within the care chain, collecting patient-related performance data, describing the tasks, reaching consensus about care domains and connect the domain of care to house-, welfare- and work domains. This expansion of integration and a more holistic view mirrors the health care reforms that were introduced in the last years. Elements such as ‘monitoring of patient judgements’ and ‘collecting patient feedback’ have been implemented less than expected, possibly due to other priorities related to external factors such as the health care reforms. Internationally, more and more attention is given to stronger patient perspectives and roles in health care and health research. Patient-oriented research is an emerging movement in Canada, the United States and the United Kingdom [23,24,25]. Recently, an international standard for patient-centered outcome measures was developed [26]. Overall, the Stroke Knowledge Network Netherlands advised their members to pay more attention to the patient perspective and the use of patient-oriented research in the coming years.

For stroke patients, the most important result eventually is if quality improvement initiatives like presented in this study do result in better outcomes for patients and if their needs are better met. To monitor the outcomes of stroke care, a national benchmark containing a set of indicators (like mortality, length of stay, % of thrombolysis treatment etc) was developed by the Stroke Knowledge Network Netherlands in 2006. Almost all stroke services participate in this indicator benchmark, however collecting data from a diversity of (it)systems in practice is challenging. Nevertheless, an interesting suggestion for further research is to analyse these outcome data of the stroke services in relation to the results of this study.

This study can also be seen as an approach of quality management for integrated care. Because of its scale, the two-yearly measurements and the use of a validated model for integrated care it is a unique approach in the Netherlands. Also, in our opinion this is one of the first studies in which the development and implementation of integrated stroke care over time was benchmarked on a national level. Nevertheless, our study has some limitations. In our study the coordinator filled in the digital self-evaluation tool on behalf of the total integrated care service. It cannot be determined to what extend coordinators consulted other stakeholders in the stroke service. When multiple stakeholders complete a self-evaluation questionnaire, a consensus score could have been calculated based on the different perspectives. Furthermore, there could be a selection bias. The stroke services who are more focused on assessing their current situation and which are interested in achieving improvements could have been more willing to participate. We tried to minimize that to invite all services and offer them the same support and time. However, we think that the included 53 services do cover a large part of the country and the geographical spread was good. Additionally, the dichotomous answer category could have influenced the results since respondents were forced to choose between ‘present’ or ‘not present’ (or unknown), while implementation processes take time and could in practice also be ‘halfway’. Another limitation is that only a survey method was used. Survey question answer options may be interpreted differently by respondents. For further research, adding interviews or focus groups could be interesting to discuss different interpretations and the results of the survey.

In conclusion, the Stroke Knowledge Network Netherlands and the individual stroke services valued the use of the self-evaluation tool which helped them to gain a deeper insight in integrated stroke care development. The individual reports gave the stroke services an overview of their current situation, the option to benchmark with others and gave suggestions for improvement. It was important to be aware of the development process of their service which helped to focus improvement. In this way the tool and model helps to more objectively discuss the current situation and possible steps in the future with their care and welfare partners. Therefore, stroke services should evaluate their aims and goals of the past year. It is recommended to stroke services to prioritize yearly their activities on basis of the phase where the stroke service is positioned in and they also need to consider the external developments in their activity plan. The results can also be used in quality policy reports and be used for local or national improvement projects. It can help to identify best practices and disseminate knowledge about these practices. As a next step, the Stroke Knowledge Network Netherlands is developing currently a peer to peer audit system for stroke services. The goals of these audits are to stimulate learning and exchange of knowledge by using a pre-established transparent framework. This framework will be based on the Development Model for Integrated Care, on the present care standards for stroke and the existing benchmark indicators for stroke. In this way it is expected that stroke services will have multiple ingredients to further improve integrated care for the benefit of stroke patients in the coming years.
